# Facile Synthesis of MXene-Ti_3_C_2_/Co Nanosheet Hydrogel Sensor with the Assistance of a Smartphone for On-Site Monitoring of Glucose in Beverages

**DOI:** 10.3390/molecules28135075

**Published:** 2023-06-28

**Authors:** Ziling Li, Tiantian Lei, Ting Pei, Keyan Chen, Zhidong Zhao, Manman Wang, Yu He

**Affiliations:** 1 School of Public Health, North China University of Science and Technology, Tangshan 063210, China; liziling02018@163.com (Z.L.); kychen1107@163.com (K.C.); zhaozhidong818@163.com (Z.Z.); 2 Ministry of Education Key Laboratory for the Synthesis and Application of Organic Functional Molecules, College of Chemistry and Chemical Engineering, Hubei University, Wuhan 430062, China; 13971096032@163.com (T.L.); 15071497176@163.com (T.P.)

**Keywords:** MXene-Ti_3_C_2_/Co NSs, peroxidase-like activity, glucose, hydrogel, point-of-care testing

## Abstract

A one-step cobaltous chloride (CoCl_2_) molten salt method was employed to prepare multilayer MXene-Ti_3_C_2_/Co materials with further ultrasonic treatment to acquire single-layer MXene-Ti_3_C_2/_Co nanosheets (NSs). MXene-Ti_3_C_2_/Co NSs were characterized, and their enzyme-like activities were investigated. Under the catalysis of MXene-Ti_3_C_2_/Co NSs, 3,3′,5,5′-tetramethylbenzidine (TMB) could be oxidized by H_2_O_2_, with the color changing from colorless to blue. The affinity of MXene-Ti_3_C_2_/Co NSs to H_2_O_2_ and TMB was better than that of nanozymes reported in previous studies. The MXene-Ti_3_C_2_/Co NSs were used for the colorimetric determination of H_2_O_2_/glucose, with limits of detection (LODs) of 0.033 mM and 1.7 μM, respectively. MXene-Ti_3_C_2_/Co NSs embedded in sodium alginate (SA) hydrogel were used to construct a sensor platform. The digital pictures combined with a smartphone-installed app (color recognizer) could be used to analyze RGB values for colorimetric detection of glucose in beverages. This point-of-care testing platform has the advantages of cost-effectiveness and good transferability, with the potential to realize quick, intelligent and on-site detection.

## 1. Introduction

Glucose is the most important energy source and metabolic intermediate of the human body and is widely present in various food products and beverages [1]. Human consumption of sugar-containing food can induce related disease, for example, obesity, kidney problems, tooth decay, diabetes mellitus and heart disease [2,3,4,5]. Diabetes caused by metabolism disorders of glucose afflicts millions of people in the world. Usually, the concentration of glucose in human blood ranges from 3.8 to 6.9 mM. When the concentration of glucose is lower than 2.8 mM after no eating or following exercise, the person is considered to be hypoglycemic. In particular, a person with diabetics should control their concentration of blood glucose below 10 mM according to the American Diabetes Association [6]. Considering that glucose in the human body is mainly derived from food products and beverages, the evaluation of the glucose concentration in food products and beverages is significant to effectively control the intake of glucose and guarantee transparency regarding dietary risks. So far, a variety of analytical techniques for glucose detection have been established, such as colorimetric [7,8], electrochemical [9,10], chemiluminescence [11,12], mass spectrometry [13,14], fluorescence [15,16] and surface-enhanced Raman scattering [17,18] methods. Among these methods, as a rapid, specific and intuitive method, commercial glucose detection kits have been widely used, involving two consecutive enzyme reactions. Unfortunately, natural enzymes have intrinsic drawbacks such as easy denaturation, difficult preparation and high cost, which hinder the further development of this method [19,20].

Developing nanoenzymes instead of natural enzymes is a promising strategy to overcome the limitations of natural enzymes. Compared with natural enzymes, nanozymes offer advantages such as controlled synthesis at low cost, tunability in catalytic activities and high stability against stringent conditions [21,22,23]. With the rapid development of nanomaterials, a variety of inorganic nanomaterials have attracted considerable attention in the field of catalysis. Since inorganic magnetic oxide (Fe_3_O_4_) was reported to exhibit peroxidase-like activity in 2007, a large number of inorganic nanomaterials, including metal nanoparticles [24,25,26], carbon-based materials [27,28] and metal oxides [22,29], have been explored as enzyme mimics to design and build colorimetric platforms for the on-site detection of analytes. Zhao et al. prepared CeO_2_-TiO_2_ nanotubes with superior peroxidase-like activity for colorimetric detection of H_2_O_2_ and glucose [30]. Nirala et al. reported the enhancement of colorimetric detection of free cholesterol in human serum by using nanocomposites of gold nanoparticles loaded on MoS_2_ nanoribbons [31].

Ti_3_C_2_ MXene-derived nanosheets (NSs) have attracted a considerable amount of attention because owing to their unique structure and fascinating optical and electronic properties and have been expanded to a wide range of application in biosensing, catalysis, batteries and LEDs. Only a few works have reported the use of Ti_3_C_2_ NS-based nanocomposites as nanoenzymes to develop sensors for the detection of targets. Li et al. prepared MXene-Ti_3_C_2_/CuS nanocomposites as peroxidase for colorimetric analysis and detection of cholesterol [32]. Li et al. prepared MXene@NiFe-LDH as a peroxidase mimic for analysis of glutathione [33]. However, the traditional preparation of Ti_3_C_2_ NS-based nanocomposites involves etching the raw materials using HF to prepare titanium carbide nanosheets, followed by further grafting with other nanoparticles to obtain composite materials. The traditional HF etching process is dangerous, complex, time-consuming and environmentally unfriendly. Therefore, there is an urgent need to develop a safer and greener approach to prepare Ti_3_C_2_ NS-based nanocomposites.

**Figure 1:**
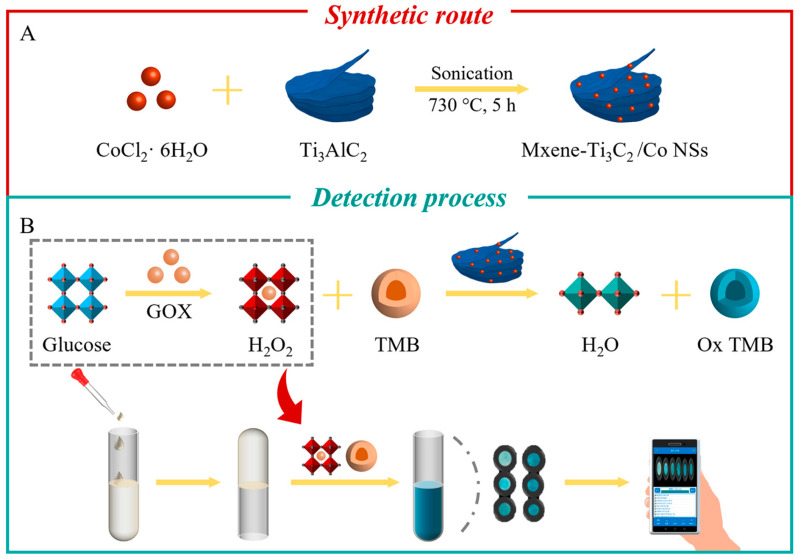
(**A**) Schematic diagram of the preparation of MXene-Ti_3_C_2_/Co NSs, (**B**) detection of H_2_O_2_ concentration and the MXene-Ti_3_C_2_/Co NSs-SA hydrogel platform for rapid colorimetric determination of glucose.

In this study, a simple one-step molten salt method was used to prepare multilayer MXene-Ti_3_C_2_/Co materials with further ultrasonic treatment to obtain single-layer MXene-Ti_3_C_2_/Co NSs (Figure 1A). The as-prepared MXene-Ti_3_C_2_/Co NSs were characterized, and their enzyme-like activities were investigated. 3,3′,5,5′-tetramethylbenzidine (TMB) could be oxidized by H_2_O_2_ under the action of peroxidase, and the color of the system gradually changed from colorless to bright blue. The MXene-Ti_3_C_2_/Co NSs exhibited superior peroxidase-mimicking activity and catalytic stability toward TMB oxidation in the presence of H_2_O_2_ to set up a sensitive and reliable colorimetric assay for the determination of H_2_O_2_ and glucose (Figure 1B). In addition, sodium alginate (SA) hydrogel, as a kind of solid-phase carrier sensor with excellent properties, such as mechanical stability, biocompatibility, porosity and biodegradation, has attracted considerable attention to be used as a stable medium for quantitative monitoring of glucose in the field. A smartphone-based hydrogel solid-phase carrier platform was established for the detection of glucose with good stability, simple and convenient operation, and high accuracy and reliability (Figure 1B).

## 2. Results and Discussion

### 2.1. Characterization of MXene-Ti_3_C_2_/Co NSs

**Figure 2:**
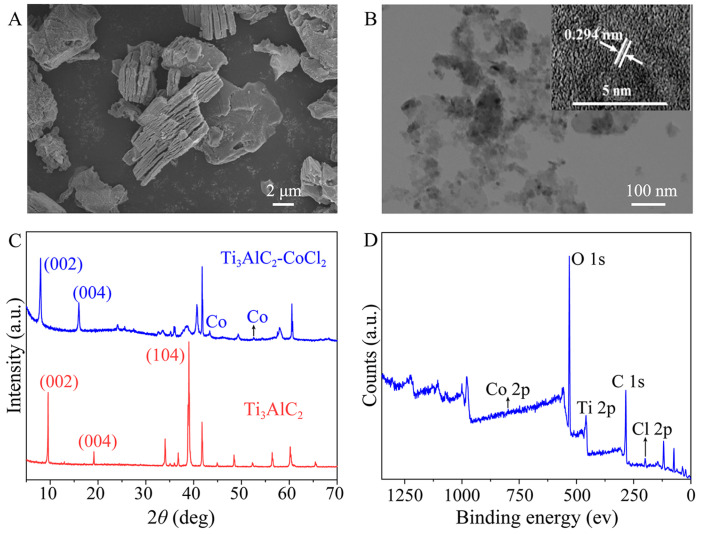
(**A**) SEM and (**B**) TEM images of MXene-Ti_3_C_2_/Co NSs (inset, upper-right: lattice fringe image); (**C**) XRD spectra of MXene-Ti_3_C_2_/Co NSs and Ti_3_AlC_2_; (**D**) survey XPS spectra of MXene-Ti_3_C_2_/Co NSs.

The MXene-Ti_3_C_2_/Co NSs were prepared by the molten salt method combined with further ultrasonic treatment. SEM images of Ti_3_AlC_2_ before and after CoCl_2_ etching are shown in  Figure S1 and Figure 2A. Compared with Ti_3_AlC_2_ before CoCl_2_ etching, the raw material Ti_3_AlC_2_ had an obvious layered structure with metal particles attached after CoCl_2_ etching. The TEM image clearly shows that the Co nanoparticles attached to the MXene-Ti_3_C_2_ NSs (Figure 2B). According to the HRTEM image (Figure 2B, inset, upper-left), the lattice spacing of MXene-Ti_3_C_2_/Co NSs was 0.294 nm. The morphology and size of MXene-Ti_3_C_2_/Co NSs were characterized by SEM and TEM, which preliminarily proved that MXene-Ti_3_C_2_/Co NSs were successfully synthesized. The XRD spectrum exhibited a peak of 2θ = 39° corresponding to position (104) of Ti_3_AlC_2_ after CoCl_2_ etching, which disappeared compared with Ti_3_AlC_2_, and the peaks corresponding to positions (002) and (004) changed significantly (Figure 2C). These results revealed that MXene-Ti_3_C_2_/Co NSs had a two-dimensional layered structure and that the Al layer in Ti_3_AlC_2_ was successfully etched by CoCl_2_, consistent with the SEM spectrum results. Next, in order to prove the elemental composition and chemical bond contained in MXene-Ti_3_C_2_/Co NSs, X-ray photoelectron spectroscopy (XPS) analysis was carried out. The full XPS spectrum showed five characteristic peaks: including Co 2p (798.0 eV), O 1s (530.9 eV), Ti 2p (458.9 eV), C 1s (284.5 eV) and Cl 2p (198.9 eV) (Figure 2D). The Co 2p spectra showed two deconvolution peaks were ascribed to Co 2p_3/2_ and Co 2p_1/2_, respectively; the Ti 2p spectra showed four characteristic deconvolution peaks corresponding to Ti-O 2p, Ti-Cl 2p and Ti-C 2p, respectively; the C 1s spectra contained a peak corresponding to C-C 1s; and the O 1s spectra showed a deconvolution peak corresponding to Ti-C-Ox (Figure S2A–D). These results prove that MXene-Ti_3_C_2_/Co NSs were successful synthesized.

### 2.2. Study of Peroxidase-like Activity of MXene-Ti_3_C_2_/Co NSs

**Figure 3:**
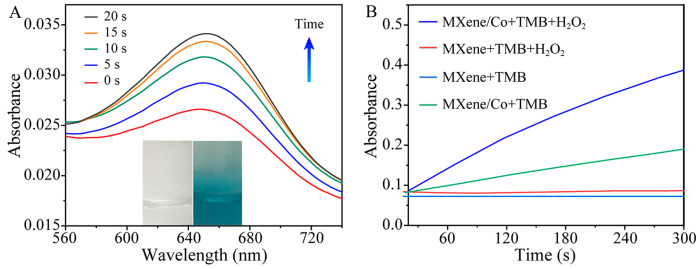
(**A**) Time-dependent UV-vis spectra (inset: photos of the solution before (left) and after (right) reaction); (**B**) time-dependent absorbance changes of TMB at 652 nm for different reaction systems in HAc-NaAc buffer (pH = 3.5) at room temperature.

The effect of buffer solution and pH on the catalytic performance was investigated in the pH range of 2–12 (Figure S3). When the pH was 3.5, MXene-Ti_3_C_2_/Co NSs showed the best catalytic performance. Therefore, HAc-NaAc (10 mM, pH 3.5) was chosen as the buffer solution. The peroxide-like activity of MXene-Ti_3_C_2_/Co NSs was investigated in the presence of H_2_O_2_ and TMB. In HAc-NaAc buffer solution (pH 3.5), TMB was difficult to oxidize with H_2_O_2_ to blue ox-TMB. However, after the addition of MXene-Ti_3_C_2_/Co NSs, the time-dependence of the absorbance of the UV-vis spectrum at 652 nm with respect to the MXene-Ti_3_C_2_/Co NSs + H_2_O_2_ + TMB ternary system was observed, as shown in Figure 3A. The absorbance gradually increased, and the color of the solution changed from colorless to blue over time, which indicates that MXene-Ti_3_C_2_/Co NSs can accelerate the reaction process between H_2_O_2_ and TMB. Furthermore, Figure 3B shows that MXenes exhibited neither oxidase-like activity nor peroxidase-like activity and could neither oxidize TMB nor catalyze the H_2_O_2_ + TMB system. Moreover, MXene-Ti_3_C_2_/Co NSs exhibited weak oxidase-like activity and strong peroxidase-like activity, indicating that they could catalyze the oxidation of TMB with H_2_O_2_ and that the UV absorbance of the MXene-Ti_3_C_2_/Co NSs + H_2_O_2_ + TMB system had a good linear relationship with the reaction time. Therefore, the application of MXene-Ti_3_C_2_/Co NSs is feasible to simulate peroxidase in H_2_O_2_ + TMB systems as a colorimetric senor.

### 2.3. Kinetic Studies and Catalytic Mechanism of MXene-Ti_3_C_2_/Co NSs

In order to study the activity of MXene-Ti_3_C_2_/Co NSs as peroxidase-like enzymes, the steady-state kinetics of MXene-Ti_3_C_2_/Co NS catalytic substrate H_2_O_2_ + TMB were discussed. First, the concentrations of H_2_O_2_ and TMB were guaranteed in an appropriate range from 0 mM to 1.0 mM. Relevant kinetic results could be obtained by changing the concentration of one substrate while other substrates were maintained at a constant concentration (Figure S4A,C). Then, a graph of the double reciprocal function of substrate concentration and the initial rate (1/*V* = (*K_m_*/*V_max_*) × (1/[S]) + 1/*V_max_*) was generated according to the Michaelis–Menten equation, where *V* represents the initial reaction rate, *V_max_* is the maximum reaction rate, *K_m_* is the Michaelis constant and can measure the affinity between enzyme and substrate and [S] represents the concentration of the substrate (Figure S4B,D). According to the double reciprocal diagram, the *K_m_* of H_2_O_2_ was calculated as 0.095 mM, while the *K_m_* of TMB was 0.107 mM. A comparison of MXene-Ti_3_C_2_/Co nanoenzymes with other nanoenzymes is shown in Table 1. Compared with other as-reported works, the *K_m_* values of H_2_O_2_ and TMB calculated in this work were lower, indicating that the affinity of MXene-Ti_3_C_2_/Co NSs with substrate H_2_O_2_ and TMB was significantly better than that of many previously reported nanoenzymes [34,35,36,37].

In addition, MXene-Ti_3_C_2_/Co NSs has two possible mechanisms as a peroxidase-like enzyme to catalyze an H_2_O_2_ + TMB substrate. The first is that the enzyme promotes electron transfer, and the other is that the enzyme catalyzes the generation of ·OH to further improve oxidation. Cyt C could be used as a standard reagent to judge the capacity of electron acceptance. This kind catalytic mechanism of a MXene-Ti_3_C_2_/Co NSs + H_2_O_2_ + TMB system could be verified by introducing cytochrome c (Cyt C), which exhibits absorption peaks at 520 nm and 550 nm (Figure S5). If Cyt C were oxidized, losing electrons, the characteristic absorption peaks at 520 nm and 550 nm would disappear, with a new absorption peak appearing at 530 nm. When Cyt C and MXene-Ti_3_C_2_/Co NSs were incubated in the dark for 48 h, two characteristic peaks of UV were still detected at 520 nm and 530 nm; thus, this catalytic mechanism can be ruled out. Terephthalic acid (TA), which easily reacted with ·OH to generate 2-hydroxyl terephthalic acid with a unique fluorescence at 435 nm, was introduced as a fluorescence probe to track ·OH. As shown in Figure S6, the fluorescence intensity of the TA + H_2_O_2_ system increases continuously with the increase in the MXene-Ti_3_C_2_/Co NSs, which indicates that the ·OH produced by the TA + H_2_O_2_ system under acidic conditions constantly increased. Therefore, the catalytic system complies with heterogeneous Fenton catalytic reactions.

### 2.4. Kinetic Studies and Catalytic Mechanism of MXene-Ti_3_C_2_/Co NSs

**Figure 4:**
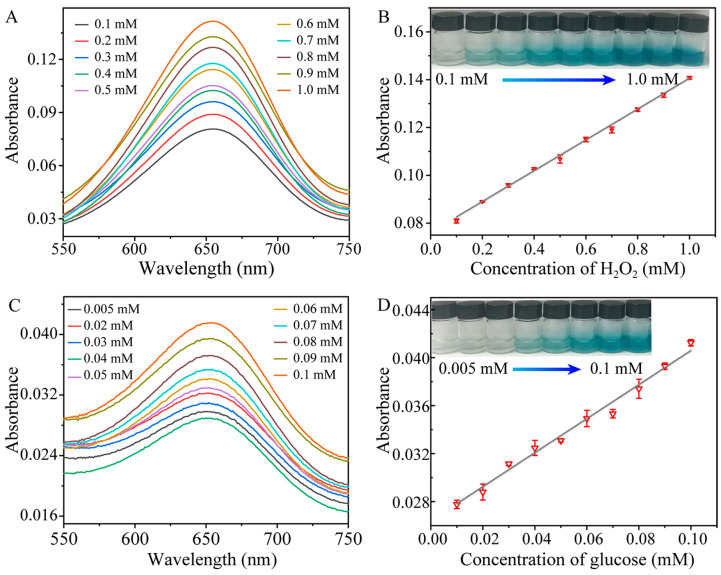
(**A**) UV-vis spectrum of the catalytic wave system with different concentrations of H_2_O_2_; (**B**) linear calibration curve of absorbance and H_2_O_2_ concentration of the system (inset: color changes of the solution with different concentrations of H_2_O_2_); (**C**) UV-vis spectrum of the catalytic system with different concentrations of glucose; (**D**) linear calibration curve of the absorbance and glucose concentration of the system (inset: color changes of the solution with different concentrations of glucose).

Figure 4A,B show the H_2_O_2_ detection performance of our sensing system using MXene-Ti_3_C_2_/Co NSs as peroxidase-like enzymes. As shown in Figure 4A, when the concentration of H_2_O_2_ increased regularly in an appropriate range, the absorbance of the ternary MXene-Ti_3_C_2_/Co NSs + H_2_O_2_ + TMB system at 652 nm also gradually increased. Figure 4B shows that the absorbance of the system had a good linear relationship with the H_2_O_2_ concentration, ranging from 0.1 mM to 1.0 mM with a calibration function of A = 0.064C + 0.076 (C was the concentration of H_2_O_2_ in mM), and the lowest detected concentration was 0.033 mM. MXene-Ti_3_C_2_/Co NSs were used to catalyze the substrate of TMB, which was ultimately oxidized into oxTMB, with its color changing from colorless to bright blue (Figure 4B, inset). Figure 4C shows that the absorbance of the system at 652 nm increased regularly with the concentration of glucose, gradually increasing from 0.005 mM to 0.1mM. The UV-vis absorbance of the system at 652 nm had an excellent linear relationship with the concentration of glucose from 0.005 mM to 0.1 mM, with a calibration function of A = 0.573C + 0.071 (where C represents the concentration of glucose in mM) and a detection limit (LOD) of 1.7 μM (Figure 4D). Furthermore, as the concentration of glucose gradually increased, the color of the MXene-Ti_3_C_2_/Co NSs + glucose + GOX + TMB system gradually changed from colorless to bright blue (Figure 4D, inset). A comparison of the MXene-Ti_3_C_2_/Co NS-based colorimetric method with other colorimetric methods is shown in Table S1. The proposed approach provided a comparable or even wider linear range and lower LOD than other colorimetric methods, which indicates that the as-prepared MXene-Ti_3_C_2_/Co NSs have practical value [38,39,40,41,42].

### 2.5. Point-of-Care Testing (POCT) for Glucose

**Figure 5:**
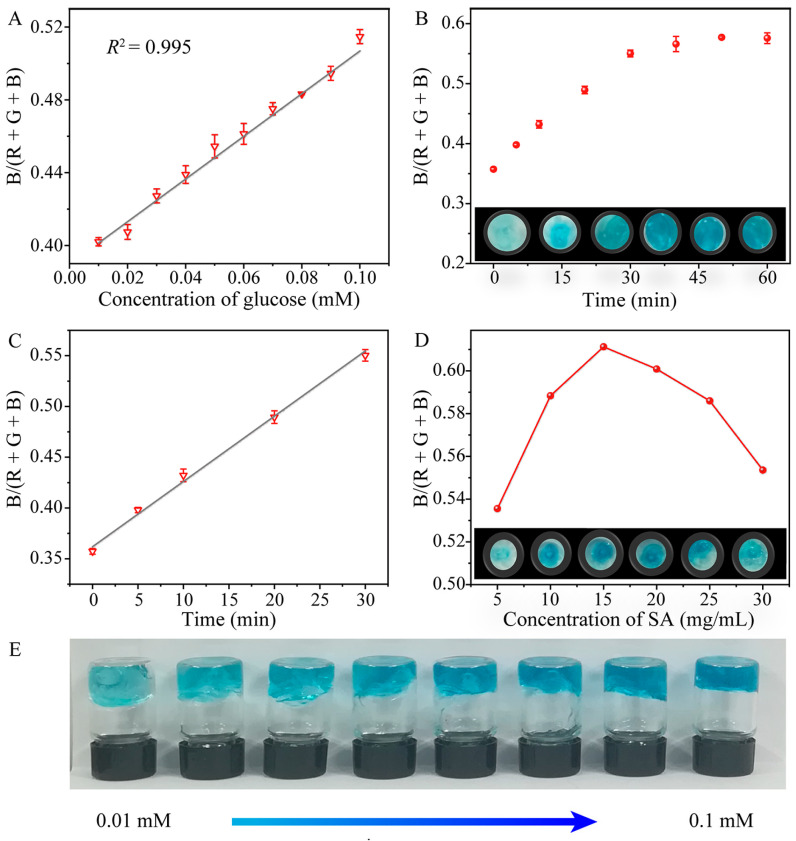
(**A**) Dose–response curves of the SA hydrogel detection platform detection with linear calibration plots for glucose; (**B**) effect of incubation time on color development; (**C**) linear calibration of color intensity against incubation time; (**D**) the influence of SA concentration on color rendering; (**E**) photographs of the SA hydrogel detection platform with different concentrations of glucose.

Most sensors developed for glucose determination are operated in solution, still requiring complicated procedures and large instruments to acquire and transfer the signals. In this work, Ti_3_C_2_ MXene/Co NSs were embedded in SA hydrogel to construct a Ti_3_C_2_ MXene/Co NS-SA hydrogel sensor platform. SA, a low-cost and biocompatible polymer, was utilized as a scaffold to increase the surface area and dispersion of the highly active catalytic centers of the Ti_3_C_2_ MXene/Co NS. Then, images of MXene-Ti_3_C_2_/Co NSs-SA hydrogel detection platform with different concentrations of glucose were imported smartphone software (color recognizer) for RGB value analysis, and the relationship between glucose concentration and color was explored, realizing the intelligent detection of glucose. It is well known that any color composed of red (R), green (G) and blue (B) has corresponding R, G and B values. Therefore, the intensity of color signal can be calculated as B/(R + G + B). Through calculation and analysis, the color intensity of the hydrogel detection platform containing different concentrations of glucose showed a good linear relationship with glucose concentration from 0.01 to 0.1 mM. The calibration curve was I = 1.172C + 0.0391 (C represents glucose concentration in mM), and the detection limit was 0.001 mM (Figure 5A). The sum of square error (SSE), mean square error (MSE) and adjusted R square were 0.0106, 0.00106 and 0.984, respectively. Furthermore, the influence of reaction time and SA concentration on the color rendering of the hydrogel detection platform for glucose concentration was explored. Figure 5B shows that the color intensity almost reached the maximum when the reaction time reached 30 min. Continuous reaction had no obvious influence on the color intensity of glucose detected by the hydrogel platform. Therefore, 30 min can be used as the optimal incubation time of glucose detected by the hydrogel platform. The linear relationship between color intensity and time is shown in Figure 5C. In addition, as shown in Figure 5D, the optimal concentration of SA was 20 mg mL^−^^1^. While the concentration of glucose gradually increased, the color of the SA hydrogel detection platform gradually changed from colorless to dark blue (Figure 5E). The digital pictures combined with a smartphone-installed app (color recognizer) can be used to analyze RGB values in order to develop a convenient method for colorimetric detection of glucose. This POCT platform with the advantages of low price, convenient operation and good transferability can be used for quick and cost-effective detection on site.

### 2.6. Selectivity for Glucose

Selectivity plays an important role in evaluating the practicality of the method. To explore the selectivity of the system, UV-vis spectra were used to measure the absorbance of the system detecting different sugars, such as glucose, fructose, maltose and sucrose. The results show that the system obviously responded to glucose, while the absorbance of the system detecting other sugars was not significantly changed, indicating that the system has excellent specificity for the detection of glucose and can be successfully applied for the detection of glucose concentration (Figure S7).

### 2.7. Real Sample Detection of Glucose

Reduced sugar intake is advocated for people attempting to lose weight and maintain health, resulting in the prevalence of sugar-free beverages in the market. Since their sugar contents can be ignored, we selected three popular sugar-free beverages in the market as actual samples. A total of 495 sample were collected to obtain data, and the reproducibility was evaluated by relative standard deviation (RSD). Based on the established detection system, the standard addition method was used to detect the glucose content. Table 2 shows that the recovery of glucose was distributed between 94.28% and 107.89%, and the range of RSD was 1.7–5.4%, indicating that the detection platform constructed in this work had good precision and can be widely used for the detection and analysis of glucose in actual samples.

## 3. Materials and Methods

### 3.1. Materials

Ti_3_AlC_2_ was purchased from Forsman Technology (Beijing) Co., LTD (Beijing, China). CoCl_2_·6H_2_O, H_2_O_2_ and sodium acetate were purchased from Sinopharm Chemical Reagent Co., Ltd. (Shanghai, China). Glucose and TMB were purchased from Aladdin Reagents Co., Ltd. (Shanghai, China). Acetic acid was purchased from Tianjin Tianli Chemical Reagent Co., Ltd. (Tianjin, China). Glucose oxidase (GOX), Tris-HCl, sodium alginate (SA) and terephthalic acid were purchased from Shanghai Macklin Biochemical Co., Ltd. (Shanghai, China). All chemical reagents were of analytical grade and used without further purification.

### 3.2. Instruments

Fluorescence spectra were evaluated by an LS55 fluorescence spectrometer (Perkin Elmer, Waltham, MA, USA). UV-vis absorption spectra were collected with a Lambda 35 UV-visible spectrometer (Perkin Elmer, Waltham, MA, USA). X-ray photoelectron spectroscopy (XPS) was measured with an Escalab 250Xi spectrometer (Thermo Fisher Scientific, Waltham, MA, USA). X-ray diffraction (XRD) was recorded with a D8 DISCOVER diffractometer (Bruker, Bremen, Germany). Transmission electron microscopic (TEM) patterns were obtained by a TecnaiG20 transmission electron microscope (FEI, Hillsboro, AL, USA). High-resolution transmission electron microscopic (HRTEM) images were observed using a JEM-2100 UHR (JEOL, Tokyo, Japan). Scanning electron microscopic (SEM) images were taken with a Zeiss SIGMA field-emission scanning electronic microscope (Carl Zeiss, Jena, Germany). An SX-4-10 box electric resistance furnace was purchased from Beijing Yongguangming Medical Instrument Factory (Beijing, China). An RZ10 medical centrifuge was purchased from Changsha Ordinary Instrument Co., Ltd. (Changsha, China).

### 3.3. Preparation of MXene-Ti_3_C_2_/Co NSs

Amounts of 0.5 g of Ti_3_AlC_2_ and 3.5 g of CoCl_2_·6H_2_O were weighed and placed in a mortar, mixed evenly and ground for 10 min. The pulverized mixture was transferred to a porcelain crucible with a volume of 50 mL and calcined at 730 °C for 1 h. After cooling and quenching, 20 mL of hydrochloric acid solution (3.0 M) was added to the powder and stirred continuously for 24 h. Next, the precipitate was washed with deionized water in a centrifuge until the supernatant pH was 7. Finally, the precipitates were collected and dried in vacuum at 70 °C for 12 h.

Then, 100 mg of the dried powder was dispersed in 25 mL deionized water with ultrasound for 5 h. The suspension was centrifuged at 5000 r for 8 min to remove precipitation; then, the supernatant was centrifuged at 9500 r for 30 min. Finally, MXene-Ti_3_C_2_/Co NSs were collected and dispersed in 5 mL deionized water for subsequent use.

### 3.4. Preparation of Hydrogel

An amount of 0.1 g of SA power was dissolved in 5 mL of Tri-HCl buffer (0.01 M) with 100 µL of MXene-Ti_3_C_2_/Co NSs and 10 µL 500 U mL^−1^ GOX. Then, MXene-Ti_3_C_2_/Co NS-SA hydrogel was formed with 5 mg mL^−^^1^ drops of CaCl_2_ solution.

### 3.5. Detection of Glucose and H_2_O_2_

The detailed steps for detection of H_2_O_2_ concentration are as follows: 10 µL of TMB (0.1 M), 30 µL of MXene-Ti_3_C_2_/Co NSs and 10 µL of H_2_O_2_ with different concentrations were added to HAc-NaAc buffer solution (pH = 3.5) in turn and reacted at room temperature for 5 min. The UV absorbance at 652 nm was recorded by a UV spectrophotometer.

The steps of glucose concentration detection are as follows: 10 μL of GOX (500 U mL^−^^1^) and 10 µL of glucose solution with different concentrations were successively introduced into 80 μL of Tris-HCl buffer (0.01 M) and incubated at 37 °C for 40 min. Then, 10 µL of TMB, 30 µL of MXene-Ti_3_C_2_/Co NSs and 860 µL of HAc-NaAc were added to the reacted mixture in turn and incubated at room temperature for 5 min. Finally, the UV absorbance at 652 nm was measured by UV spectrophotometer.

### 3.6. Intelligent Detection of Glucose by SA Hydrogel Sensor

The intelligent detection steps for glucose are as follows: 10 µL 500 U mL^−1^ GOX and 10 μL of glucose solution with different concentrations were mixed in 80 μL of Tri-HCl buffer (0.01 M) and incubated at 37 °C for 40 min. The obtained mixed solution, 60 μL of HAc-NaAc buffer (pH = 3.5) and 10 μL TMB (0.1 M) were successively added into SA hydrogel to react for 30 min. Color photographs of hydrogels with different glucose concentrations were taken by a smartphone. The color signal (RGB value) including a red channel (R), blue channel (B) and green channel (G) was obtained by a smartphone application (color recognizer). The linear relationship between the B/(R + G + B) value and the concentration of glucose was obtained.

### 3.7. Detection of Glucose in Real Samples

We selected three popular sugar-free beverages, namely Yuanqi forest bubble water, alien electrolyte water and soda water, as actual samples and analyzed their glucose contents. The beverage samples were diluted 50 times as the actual samples to be tested. Then, 70 µL of Tris-HCl buffer (0.01 M), 10 µL of GOX and 20 µL of the beverage samples were incubated at 37 °C for 40 min. Then, 845 µL of HAc-NaAc buffer (pH = 3.5), 25 µL of TMB and 30 µL of MXene-Ti_3_C_2_/Co NSs were introduced into the mixture to cultivate for 5 min. The absorbance of the solution was determined by a UV spectrometer.

## 4. Conclusions

In conclusion, MXene-Ti_3_C_2_/Co NSs with excellent dispersion were prepared by efficient molten salt method. MXene-Ti_3_C_2_/Co NSs were successfully applied in a UV detection system for the determination of trace concentrations of H_2_O_2_ and glucose due to their excellent peroxidase-like activity. In order to realize the rapid detection of glucose concentration, a hydrogel detection platform based on MXene-Ti_3_C_2_/Co NSs was constructed in combination with a smartphone analyzer by recording changes in RGB value with a smartphone app, and the results were consistent with those of the UV spectrophotometer. Compared with existing glucose monitoring approaches, the *K_m_* values of H_2_O_2_ and TMB in our proposed method were lower, indicating that the affinity of MXene-Ti_3_C_2_/Co NSs with substrate H_2_O_2_ and TMB was significantly better than that of many previously reported nanoenzymes. Owing to the simplicity of the method, the sample throughput can be easily increased, making it potentially useful for on-site monitoring of glucose.

## Figures and Tables

**Table 1:** Comparison of the Michaelis–Menten constants (*K_m_*) and maximum reaction rates (*V_max_*) among several nanomaterials and enzymes.

Sample	*K_m_* (mM)		*V_max_* (10^−8^M·S^−1^)		Reference
	TMB	H_2_O_2_	TMB	H_2_O_2_	
HRP	0.275	0.214	1.24	2.46	[34]
Ti_3_C_2_ nanosheets	0.433	0.015	12.1	1.44	[35]
Ala-Ti_3_C_2_	0.281	0.012	10.37	1.43	[35]
MXene/CuS	0.072	2.08	4.63	6.34	[36]
GO-Fe_3_O_4_	0.43	0.71	13.08	5.31	[37]
MXene-Ti_3_C_2_/Co	0.107	0.095	2.27	2.04	This work

**Table 2:** Analytical results of glucose detection in sugar-free beverages.

Sample	Spiked (µM)	Found (µM)	Recovery (%)	RSD (%)
Wonki Fores	25	25.23	100.92	1.8
	50	47.78	95.56	4.5
	75	80.92	107.89	2.3
Alien electrolyte	25	24.34	97.36	5.4
	50	52.29	104.58	3.1
	75	75.91	101.21	1.7
Soda	25	26.49	105.96	2.4
	50	48.39	96.78	3.8
	75	70.71	94.28	2.1

## Data Availability

Not applicable.

## Supplementary Materials

The following supporting information can be downloaded at: https://www.mdpi.com/article/10.3390/molecules28135075/s1. Figure S1: SEM image of Ti_3_AlC_2_ before CoCl_2_ etching; Figure S2: XPS spectrum of MXene-Ti_3_C_2_/Co NS: (A) C 1s, (B) O 1s, (C) Ti 2p, (D) Co 2p; Figure S3: The influence of pH on the catalytical performance of MXene-Ti_3_C_2_/Co NSs; Figure S4: (A,C) Steady-state kinetic curves with the Michaelis−Menten equation and (B,D) the corresponding double reciprocal plots with the Lineweaver−Burk method for the MXene-Ti_3_C_2_/Co nanocomposites. (A,B) Concentration of TMB is fixed at 1.0 mM, whereas that of H_2_O_2_ varies; (C,D) concentration of H_2_O_2_ is 1 mM, whereas the TMB concentration changes; Figure S5: UV-vis spectra of Cyt C before and after interacting with Cu NCs/Ti_3_C_2_ NSs; Figure S6: Fluorescence spectra of the interaction among TA + H_2_O_2_ and MXene-Ti_3_C_2_/Co nanosheets with different concentrations; Figure S7: Selectivity for glucose; Table S1: Comparison of different colorimetric methods for glucose detection.

## Sample Availability

Samples of the compounds are available from the authors.
