# Supplementary material for: Facile Synthesis of MXene-Ti3C2/Co Nanosheet Hydrogel Sensor with the Assistance of a Smartphone for On-Site Monitoring of Glucose in Beverages
Source: Molecules. 2023 Jun 28;28(13):5075. doi: 10.3390/molecules28135075 (PMC10343734; doi:10.3390/molecules28135075)
Supplement: Supplementary file 1 [file molecules-28-05075-s001.zip › molecules-2456281-supplementary.pdf]

Supporting Information

Facile Synthesis of MXenes-Ti_3C_2/Co Nanosheets-Hydrogel Sen-Sor with the Assistance of Smartphone for On-Site Monitoring of Glucose in Beverages

Ziling Li [1], Tiantian Lei [2], Ting Pei [2], Keyan Chen [1], Zhidong Zhao [1], Manman Wang [1,*] and Yu He [2,*]

[1] School of Public Health, North China University of Science and Technology, Tangshan 063210, China; liziling02018@163.com (Z.L.); kychen1107@163.com (K.C.); zhaozhidong818@163.com (Z.Z.)
[2] Ministry-of-Education Key Laboratory for the Synthesis and Application of Organic Functional Molecules, College of Chemistry and Chemical Engineering, Hubei University, Wuhan 430062, China; 13971096032@163.com (T.L.); 15071497176@163.com (T.P.)
* Correspondence: wangmanman@ncst.edu.cn (M.W.); heyu@hubu.edu.cn (Y.H.)

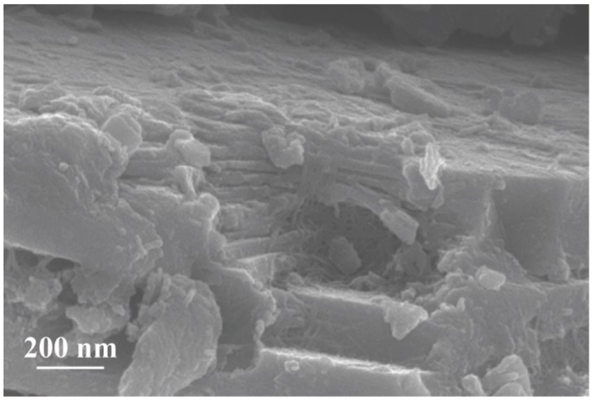

Figure S1. SEM image of Ti_3AlC_2 before $CoCl_2$ etching.

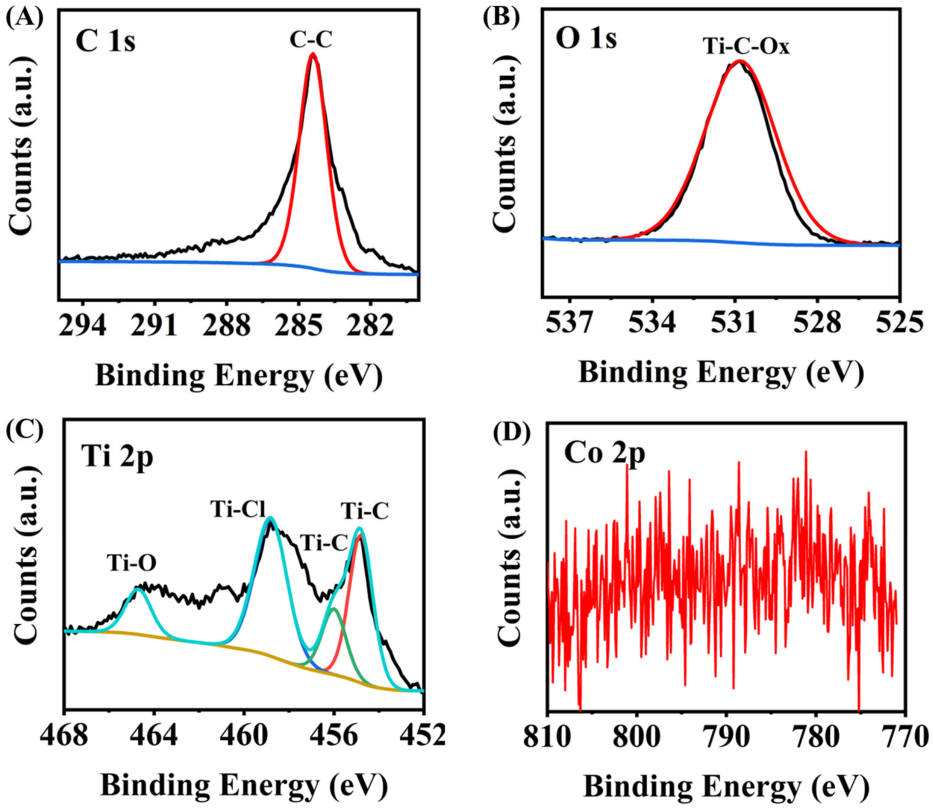

Figure S2. The XPS spectrum of MXenes-Ti_3C_2/Co NS: (A) C 1s, (B) O 1s, (C) Ti 2p, (D) Co 2p.

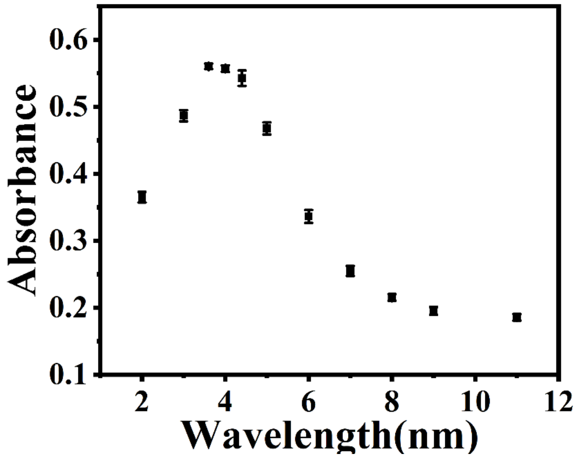

Figure S3. The influence of pH on the catalytical performance of MXene-Ti_3C_2/Co NSs.

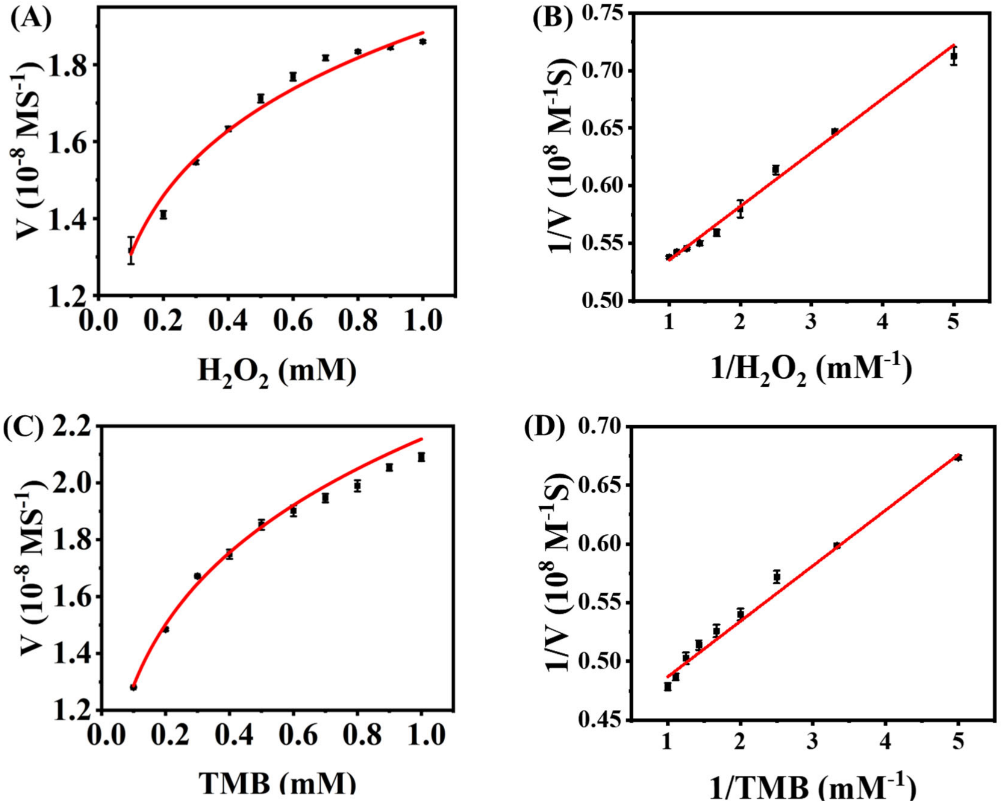

Figure S4. Steady-state kinetic curves with the Michaelis−Menten equation (A, C) and the corresponding double-reciprocal plots with the Lineweaver−Burk method (B, D) for the MXene-Ti_3C_2/Co nanocomposites. (A, B) Concentration of TMB is fixed at 1.0 mM, whereas that of H_2O_2 varies; (C, D) concentration of H_2O_2 is 1 mM, whereas the TMB concentration changes.

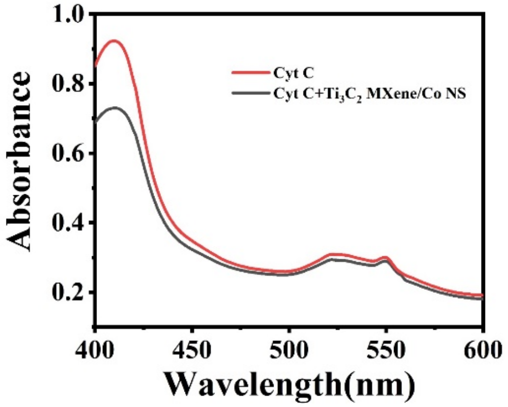

Figure S5. UV-vis spectra of Cyt C before and after interacting with Cu NCs/Ti_3C_2 NSs.

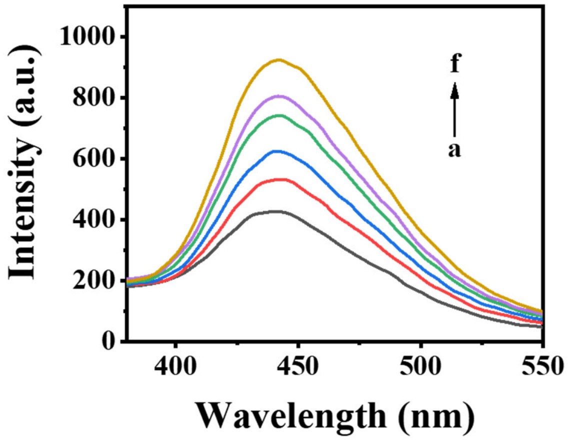

Figure S6. Fluorescence spectra for interaction among TA+H_2O_2 and MXene-Ti_3C_2/Co nanosheets with different concentration.

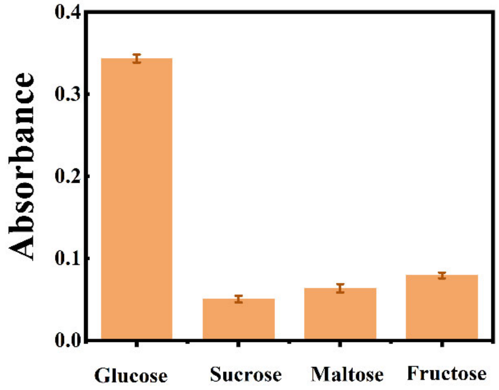

Figure S7. Selectivity for glucose.

Table S1. Comparison of different colorimetric methods for glucose detection.

Sensor	Linear Range (μM)	LOD (μM)	Reference
MnO_2-nanosheet-modified upconversion nanoparticles	0-250	3.7	[38]
Copper nanoclusters	100-2000	100	[39]
Carbon quantum dots	20-600	2.18	[40]
CuZnFeS nanocrystals	16-60	4.1	[41]
5,10,15,20-tetrakis (4-carboxyl pheyl)-porphyrin functionalized NiO nanoparticles	50-500	20	[42]
MXene-Ti_3C_2/Co NSs	5-100	1.7	This work
